# Y-mediated optimization of 3DG-PbO_2_ anode for electrochemical degradation of PFOS

**DOI:** 10.1186/s13065-023-01057-3

**Published:** 2023-10-27

**Authors:** Xiaoyue Duan, Ziqi Ning, Weiyi Wang, Yitong Li, Xuesong Zhao, Liyue Liu, Wenqian Li, Limin Chang

**Affiliations:** 1 Key Laboratory of Preparation and Applications of Environmental Friendly Materials (Jilin Normal University), Ministry of Education, Changchun, 130103 China; 2 Key Laboratory of Environmental Materials and Pollution Control, Education Department of Jilin Province, Jilin Normal University, Siping, 136000 China; 3 College of Engineering, Jilin Normal University, Siping, Jilin China

**Keywords:** Electrochemical oxidation, PbO_2_ anode, 3DG, Yttrium, PFOS

## Abstract

In our previous study, the three-dimensional graphene-modified PbO_2_ (3DG-PbO_2_) anode was prepared for the effective degradation of perfluorooctanesulfonat (PFOS) by the electrochemical oxidation process. However, the mineralization efficiency of PFOS at the 3DG-PbO_2_ anode still needs to be further improved due to the recalcitrance of PFOS. Thus, in this study, the yttrium (Y) was doped into the 3DG-PbO_2_ film to further improve the electrochemical activity of the PbO_2_ anode. To optimize the doping amount of Y, three Y and 3DG codoped PbO_2_ anodes were fabricated with different Y^3+^ concentrations of 5, 15, and 30 mM in the electroplating solution, which were named Y/3DG-PbO_2_-5, Y/3DG-PbO_2_-15 and Y/3DG-PbO_2_-30, respectively. The results of morphological, structural, and electrochemical characterization revealed that doping Y into the 3DG-PbO_2_ anode further refined the β-PbO_2_ crystals, increased the oxygen evolution overpotential and active sites, and reduced the electron transfer resistance, resulting in a superior electrocatalytic activity. Among all the prepared anodes, the Y/3DG-PbO_2_-15 anode exhibited the best activity for electrochemical oxidation of PFOS. After 120 min of electrolysis, the TOC removal efficiency was 80.89% with Y/3DG-PbO_2_-15 anode, greatly higher than 69.13% with 3DG-PbO_2_ anode. In addition, the effect of operating parameters on PFOS removal was analyzed by response surface, and the obtained optimum values of current density, initial PFOS concentration, pH, and Na_2_SO_4_ concentration were 50 mA/cm^2^, 12.21 mg/L, 5.39, and 0.01 M, respectively. Under the optimal conditions, the PFOS removal efficiency reached up to 97.16% after 40 min of electrolysis. The results of the present study confirmed that the Y/3DG-PbO_2_ was a promising anode for electrocatalytic oxidation of persistent organic pollutants.

**Supplementary Information:**

The online version contains supplementary material available at 10.1186/s13065-023-01057-3.

## Introduction

Perfluorooctane sulfonate (PFOS) is one of the typical perfluoroalkyl substances (PFASs). Due to the unique characteristics of high hydro- and lipo- phobic, good thermal and chemical stability, and excellent surfactant properties, PFOS has been broadly used in industrial applications and the manufacturing processes of daily consumer goods [1, 2]. However, PFOS is easily accumulated in water bodies due to its water solubility and strong persistence [3]. Previous reports indicate that PFOS has been widely distributed around the world and even detected in drinking water and human fluids (serum, breast milk, and urine) [4–6]. In addition, PFOS is one of the environmental endocrine-disrupting chemicals (EDCs) that might induce obesity, cardiovascular problems, cancer, and infertility [7–9]. Therefore, PFOS has posed a great potential threat to the ecological environment and human health, and it is meaningful to explore effective methods to degrade PFOS.

Because of the high energy of C-F bonds (485 kJ/mol) [10], the PFOS has high stability and is extremely resistant to biological degradation. Thus, various methods have been explored to treat PFOS, including physical adsorption [11], constructed wetlands (CWs) [12, 13], photochemical method [14, 15], electrochemical oxidation [16], sonochemical oxidation [17], hydrothermal reaction [18], beams of electron and plasma [19, 20], etc. Among these processes, the electrochemical oxidation process has attracted growing attention due to its excellent oxidation efficiency, mild reaction conditions, simple operation, and environmental compatibility [21, 22]. Shi et al. constructed a reactive electrochemical membrane system, in which 98.30 ± 0.51% of PFOS was removed through cross-flow filtration and following electrochemical oxidation processes [23]. Li et al. combined electrochemical oxidation and UV irradiation to treat PFOS, in which the PFOS removal efficiency was significantly greater than the mathematical addition of the solo electrochemical oxidation and UV irradiation systems [24]. The process of adsorption onto graphite intercalated compounds (GIC) and following electrochemical oxidation was proposed by Trzcinski and Harada, and 99% of PFOS was removed with a half-life of 15 min [25]. Yang et al. fabricated a novel Ti/Sn-Sb/SnO_2_-F-Sb anode for electrochemical oxidation of PFOS, and more than 99% of PFOS was removed after 120 min electrolysis [16]. These reports confirm that the electrochemical oxidation process can effectively degrade PFOS either alone or in combination with other technologies.

It is well known that the anode is the heart of electrochemical oxidation, which directly affects the degradation effect of organic pollutants [26, 27]. PbO_2_ has been regarded as one of the most popular anode materials because of its easy preparation, low cost, excellent electrocatalytic activity, and high stability [28, 29]. To meet the demand for high electrocatalytic performance for degrading PFOS, we developed a novel three-dimensional graphene-modified lead dioxide (3DG-PbO_2_) anode in our previous study [30]. 3DG significantly increased the electrocatalytic activity of the PbO_2_ electrode, which effectively removed PFOS with a degradation rate of 96.17% after 120 min of degradation, much higher than that of pure PbO_2_ anode (75.13%) [30]. Nevertheless, we found that the mineralization rate of PFOS was slow for the electrochemical oxidation of PFOS at the 3DG-PbO_2_ anode, only 68.58% of TOC was removed after 120 min of electrolysis. Therefore, the better electrocatalytic activity of 3DG-PbO_2_ is needed for the electrochemical oxidation of PFOS.

Rare earth elements have shown superior electrical, thermoelectric, magnetic, optical, and biological properties due to their incompletely occupied 4f electronic configuration, which have been widely used in many fields consisting of electronics, metallurgy, aerospace, photolysis, fuel cell, etc. [31–33]. Some rare earth elements have also been used to improve the activity and stability of PbO_2_ anodes. Jin et al. reported that doping Ce and polyvinylpyrrolidone (PVP) into the active layer of β-PbO_2_ significantly reduced the charge transfer resistance of the PbO_2_ electrode and enhanced the electrocatalytic oxidation performance of the PbO_2_ electrode for treating methyl orange dye wastewater [34]. Zhang and co-workers found that doping terbium not only improved the electrocatalytic activity of Ti/PbO_2_ anode, but also prolonged its service life to183 h, 14 times longer than that of Ti/PbO_2_ (13 h) [35]. Lan et al. fabricated a Yb-GO-PbO_2_ anode, which presented better activity than the pure PbO_2_ anode for the degradation of lamivudine [36]. Wang et al. reported that the electrochemical activity of different rare earth elements (La, Ce, Gd, and Er) doped PbO_2_ anodes for degradation of *p*-nitrophenol followed the order of Er-PbO_2_ > Gd-PbO_2_ > La-PbO_2_ > Ce-PbO_2_ > PbO_2_ [37]. Eu-doped PbO_2_ anode was also reported to have higher oxygen evolution overpotential, stability, and electrocatalytic activity than the pure PbO_2_ anode [38].

As one of the early rare earth metals, Yttrium (Y) has been more and more widely used to promote the activity of various catalysts. Reddy et al. prepared Cu and Y co-doped hematite (α-Fe_2_O_3_) nanostructured photoanodes, and the addition of Y not only increased the electron-hole density of the photoanode but also improved its catalytic activity [39]. Hou et al. synthesized a Y-doped titanium dioxide (nY/TiO_x_) catalyst, which showed superior catalytic activity and higher stability than the pristine TiO_2_ catalyst for propane dehydrogenation [40]. The Y^3+^ and Co^2+^ codoped LaNiO_3_ exhibited better activity than pure LaNiO_3_ in the photocatalytic degradation of Rhodamine B [41]. The electronic structure of ZnO was changed by doping Y, thereby enhancing the photoelectrochemical performance and prolonging the carrier lifetime of ZnO for water splitting [42]. Yu and coworkers reported that the incorporation of Y_2_O_3_ promoted the electrocatalytic activity of the PbO_2_ anode due to enhanced electron transfer rate and accelerated generation of reactive oxygen species [43]. Khalfaouy et al. synthesized yttrium-substituted LiMn_1 − x_Y_x_PO_4_/C cathode material for lithium-ion batteries, in which yttrium-substituting reduced the charge transfer impedance, improved the lithium-ion diffusion, and the specific discharge capacity of LiMnYPO_4_/C was improved by 14% [44]. Yittrium-doped SnO_2_-NiO nano-composite also exhibited higher specific capacitance and better reversible performance for energy storage applications [45].

Based on the above studies, we predict that Y should be a promising dopant for modifying the PbO_2_ anode, which can productively promote the electrocatalytic activity of the PbO_2_ anode owing to its unique physical and chemical properties. Besides, the ionic radius of Y^3+^ ions is much smaller than that of Pb^2+^ ions, and close to that of Pb^4+^ ions. Consequently, it can easily replace the Pb^2+^ into PbO_2_ film and its incorporation will increase the stability of the PbO_2_ film [46, 47]. Herein, in this work, the rare earth metal element of Y was selected as a dopant to be codoped into the PbO_2_ anode with 3DG (the anode was denoted by Y/3DG-PbO_2_) by the composite electrodeposition method. The effect of doping Y on the surface micromorphology, crystal structure, and elemental chemical state of the 3DG-PbO_2_ anode was analyzed by scanning electron microscopy (SEM), X-ray diffraction (XRD), and X-ray photoelectron spectroscopy (XPS). The electrochemical performance of the Y/3DG-PbO_2_ anode was investigated by linear sweep voltammetry (LSV), cyclic voltammetry (CV), electrochemical impedance spectroscopy (EIS), and Tafel. In addition, the electrocatalytic oxidation behavior of perfluorooctane sulfonate (PFOS) at Y/3DG-PbO_2_ anode was investigated intensively.

## Experimental

### Materials

Pure titanium sheets were obtained from Jinkai Co., Ltd. (Baoji, China). Y(NO_3_)_3_•6H_2_O, L-ascorbic acid, and PFOS were purchased from Energy Chemical (Shanghai, China). Pb(NO_3_)_2_, SnCl_4_•5H_2_O, SbCl_3_, Na_2_SO_4_, C_3_H_7_OH, HNO_3_, H_2_C_2_O_4_, and HCl were provided by Sinopharm (Shanghai, China). NaF and PbO were supplied by Shenyang Chemical Reagent Factory (Shenyang, China). All chemicals used were analytical reagents and used directly without any further purification. All the solutions were prepared with deionized water.

### Preparation of Y/3DG-PbO_2_ anode

The preparation process of Y/3DG-PbO_2_ anodes was followed as that of 3DG-PbO_2_-0.08 anode in our previous study [30], consisting of pretreatment, thermal deposition of SnO_2_-Sb_2_O_3_ bottom layer, electrodeposition of α-PbO_2_ intermediate layer and electrodeposition of β-PbO_2_ active layer. Different from the preparation process of 3DG-PbO_2_-0.08, the electroplating solution of the active layers of Y/3DG-PbO_2_ anodes contained 5, 15, or 30 µM Y(NO_3_)_3_. The obtained anodes were named Y/3DG-PbO_2_-5, Y/3DG-PbO_2_-15, and Y/3DG-PbO_2_-30, respectively. For morphological comparison, the pure PbO_2_, Y-PbO_2_-5, Y-PbO_2_-15, and Y-PbO_2_-30 anodes were also fabricated, whose preparation process was the same as those of 3DG-PbO_2_, Y/3DG-PbO_2_-5, Y/3DG-PbO_2_-15, and Y/3DG-PbO_2_-30 anodes, respectively except that no 3DG was added to the electroplating solution of the active layer.

### Analytical methods

A scanning electron microscope (JEOL JSM-6510) was used to characterize the surface morphology of anodes. X-ray diffractometer (PC 2500, Rigaku) equipped with Cu kα irradiation (λ = 0.154060 nm) was used to analyze the phase composition of samples with a scanning rate of 16°/ min. XPS was carried on an ESCALAB250XI X-ray photoelectron spectrometer equipped with Al Kα radiation (1486.60 eV, 150 W) to analyze the chemical state of elements on the surface of electrodes.

All the electrochemical tests including LSV, CV, EIS, and Tafel were performed on an electrochemical workstation (IVIUMSTAT, Netherlands) in 0.5 M H_2_SO_4_ solution with a standard three-electrode system. The as-prepared anodes were used as the working electrodes, a platinum sheet as the auxiliary electrode, and a saturated calomel electrode as the reference electrode. The LSV was tested in the potential range from 1 to 2.5 V with a scan rate of 50 mV/s. The CV was tested at scan rates of 20, 40, 60, 80, and 100 mV/s in a potential range of 0.5-2.0 V. The EIS was tested in a frequency range from 0.01 to 10^4^ Hz with an applied sine wave of 10 mV amplitude. The Tafel curves were obtained in a potential range from 1.0 to 1.6 V and at a scan rate of 0.166 mV/s.

The active species were measured on a Hitachi JES-FA200 electron spin resonance (ESR) spectrometer. Since the terephthalic acid (TA) can easily react with •OH radicals to form the fluorescent 2-hydroxyterephthalic acid (2-HA), it was used as a probe molecule to test the •OH radical generation of prepared anodes. The concentration of 2-HA was measured on a fluorescence spectrophotometer (Cary EclipseG9800A, Agilent, λ_ex_ = 315 nm and λ_em_ = 425 nm). The •OH radical generation experiments were conducted at a constant current density of 30 mA/cm^2^ in 100 mL of 0.75 mM TA + 0.25 M Na _2_SO_4_ + 0.5 g/L NaOH solution at a temperature of 30 °C. 0.8 mL of electrolyte was drawn at five-minute intervals and diluted 5 times with deionized water.

The concentrations of PFOS were analyzed on a liquid chromatography-mass spectrometry (LC-MS/MS, Agilent 6120) with an XBridge C18 column (4.6 mm×150 mm, 3.5 µL). Gradient mobile elution was delivered at a flow rate of 1.5 mL/min which was composed of eluent A (10 mM ammonium acid carbonate in 100% water) and eluent B (100% acetonitrile). In the initial 2.0 min, the acetonitrile gradient was increased from 5% to 95%. In 2.0–12.0 min, the acetonitrile was held at 95% for 10 min. The PFOS removal efficiency was calculated according to Eq. (1):

1 $$r=\frac{{C}_{0}-{C}_{t}}{{C}_{0}}\times 100\%$$

where *r* is the removal efficiency of PFOS, *C*_0_ is the initial PFOS concentration, and *C*_*t*_ is the PFOS concentration at a given time *t*. The TOC value of the degradation solution was measured on a TOC analyzer (Vario TOC). The TOC removal efficiency was calculated according to Eq. (2).

2 $$\eta =\frac{\text{TO}{\text{C}}_{0}-\text{TO}{\text{C}}_{t}}{\text{TO}{\text{C}}_{0}}\times 100\%$$

where *η* is the TOC removal efficiency, TOC_0_ is the initial TOC concentration of PFOS solution, and TOC_*t*_ is the TOC concentration at a given time *t*. The energy consumption (EC) on each anode was calculated to evaluate the energy efficiency of PFOS degradation according to Eq. (3) [48].

3 $$EC=\frac{\left({U}_{cell}I\right){t}_{90\%}}{V}$$

where *U*_cell_ is the average cell voltage (V), *I* is the applied current (A), *t*_(90%_) is the time for 90% PFOS removal (h), and *V* is the volume of PFOS solution (m^3^). The concentration of Pb^2+^ ions in the degradation solution was determined on an Agilent 7500 inductively coupled plasma mass spectrometer (ICP-MS).

### Electrochemical oxidation of PFOS

The electrocatalytic oxidation of PFOS was performed in 200 mL of organic glass rector with continuous magnetic stirring at a constant temperature of 30 °C. The initial PFOS concentration was 50 mg/L, 0.05 M Na_2_SO_4_ was used as the supporting electrolyte, and the applied current density was 30 mA/cm^2^. The as-prepared Y/3DG-PbO_2_ or 3DG-PbO_2_ electrode with an effective area of 3 cm × 5 cm served as the anode. The ratio of electrode surface area to working volume was 3/40 cm^2^/mL. Since only hydrogen evolution reaction occurs on the cathode, which doesn’t participate in the degradation process [49], a conductive stainless steel sheet with the same area as the anode was used as the cathode. Two electrodes were positioned vertically and parallel to each other with an inter-distance of 2 cm.

## Results and discussion

### Characterization of Y/3DG-PbO_2_ electrode

**Fig. 1:**
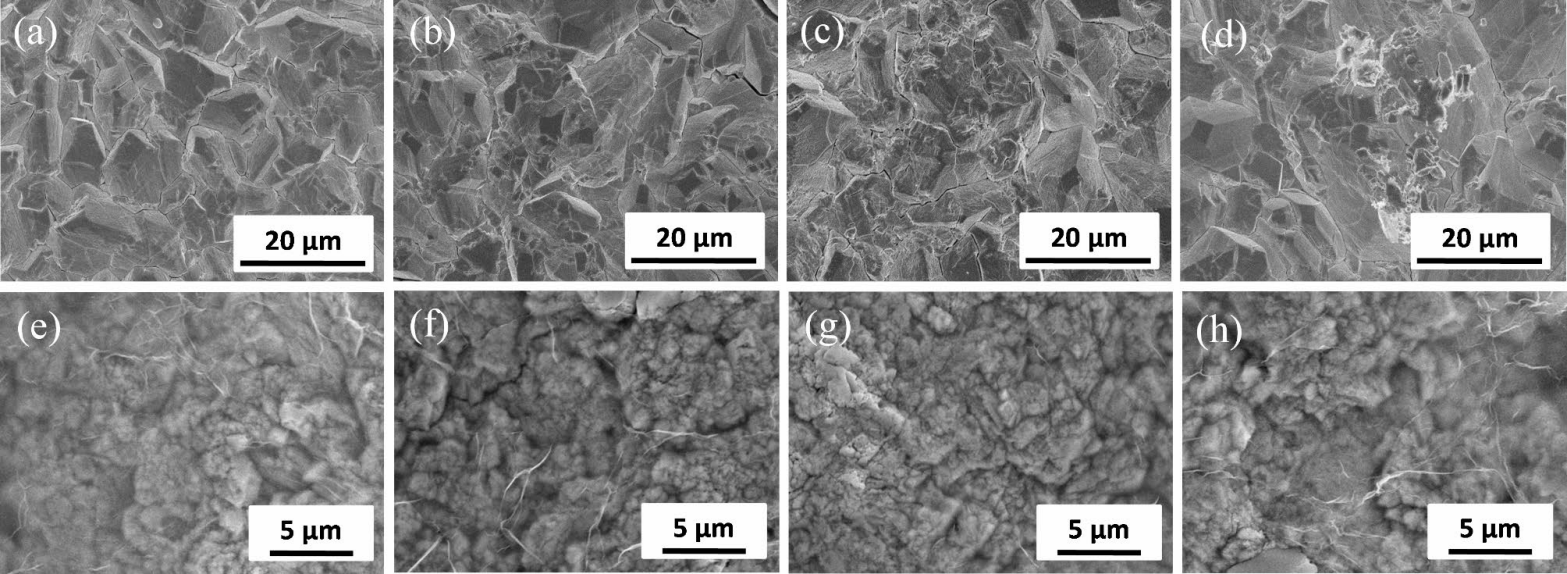
SEM images of pure PbO_2_**(a)**, Y-PbO_2_-5 **(b)**, Y-PbO_2_-15 **(c)**, Y-PbO_2_-30 **(d)**, 3DG-PbO_2_**(e)**, Y/3DG-PbO_2_-5 **(f)**, Y/3DG -PbO_2_-15 **(g)**, and Y/3DG -PbO_2_-30 **(h)** electrodes

SEM analysis was carried out to observe the surface morphology of the prepared Y/3DG-PbO_2_ anodes. For comparison, the SEM images of pure PbO_2_ and Y-PbO_2_ anodes were also presented in Fig. 1a-d. It can be observed that the surface of pure PbO_2_ electrode was composed of unevenly sized pyramidal crystals (Fig. 1a). After adding 5 or 15 µM Y^3+^, the crystal size of the β-PbO_2_ gradually decreased and the crystals were more compact (Fig. 1b and c). However, when the concentration of Y^3+^ was further improved to 30 μm, the crystal size of the β-PbO_2_ became uneven, and some crystals stacked together (Fig. 1d). Compared to the pure PbO_2_ and Y-PbO_2_ anodes, the morphology of 3DG-PbO_2_ and Y/3DG-PbO_2_ was evident, where PbO_2_ particles were well wrapped by graphene sheets (Fig. 1e-h). This indicated that a strong coupling was formed between the graphene sheets and PbO_2_ particles. It also can be seen that the crystal sizes of Y/3DG-PbO_2_ anodes were greatly smaller than those of pure PbO_2_ and Y-PbO_2_ anodes, which may be due to that the graphene sheets acted as support template for growing the PbO_2_ crystals [50]. Among three Y/3DG-PbO_2_ anodes, the Y/3DG-PbO_2_-15 anode possessed the most uniform, flat, and dense surface, which was consistent with the better surface of the Y-PbO_2_-15 anode.

**Fig. 2:**
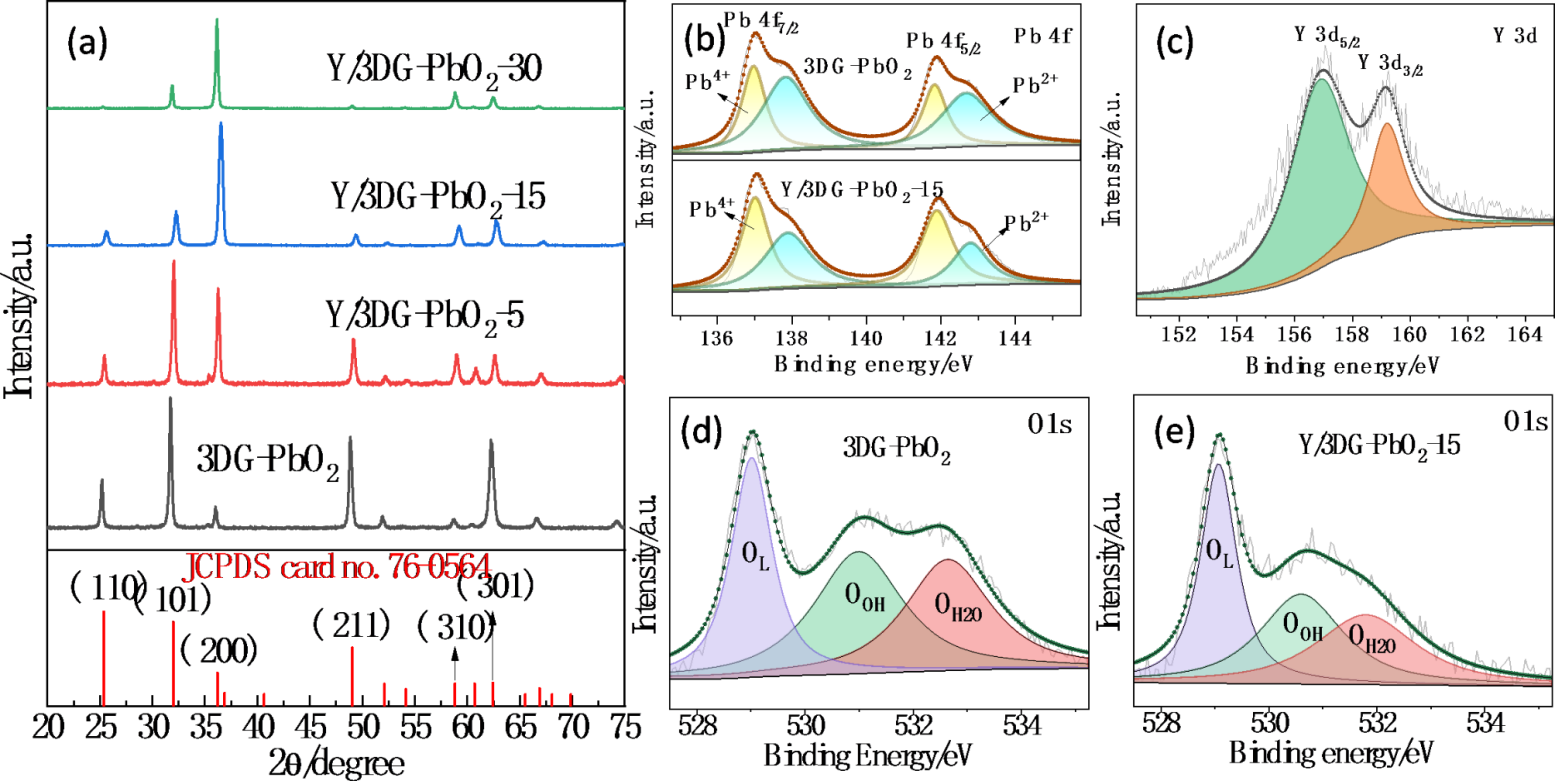
**(a)** XRD patterns of prepared anodes, XPS spectra of Pb 4f for 3DG-PbO_2_ and Y/3DG-PbO_2_-15 anodes **(b)**, Y 3d for Y/3DG-PbO_2_-15 anode **(c)**, and O 1s for 3DG-PbO_2_**(d)** and Y/3DG-PbO_2_-15 **(e)** anodes

The effect of doped Y on the crystal phase structure of the 3DG-PbO_2_ anode was investigated by XRD. Figure 2(a) shows the XRD patterns of 3DG-PbO_2_, Y/3DG-PbO_2_-5, Y/3DG-PbO_2_-15, and Y/3DG-PbO_2_-30 anodes. All the anodes presented characteristic diffraction peaks at 2θ = 25.3°, 31.9°, 36.1°, 49.1°, 58.8°, 62.5°, and 85.6°, which are well indexed to the (110), (101), (200), (211), (310), (301), and (411) planes of β-PbO_2_ crystals (JCPDS card no. 76–0564) [27, 51, 52]. This demonstrates that doping Y didn’t affect the formation of β-PbO_2_ crystals. It also can be observed from Fig. 2(a) that the diffraction peak intensity of the (200) plane of 3DG-PbO_2_ was significantly increased after doping Y, while the diffraction peak intensities of (110), (101), (211), and (301) planes were reduced. This phenomenon indicates that doping Y changed the preferred growth orientation of β-PbO_2_, which may influence the electro-catalytic activity of the 3DG-PbO_2_ anode [27].

XPS measurements were performed to investigate the chemical states of elements on the surface of 3DG-PbO_2_ and Y/3DG-PbO_2_-15 anodes. As shown in Fig. 2(b), both of the high-solution XPS spectra of 3DG-PbO_2_ and Y/3DG-PbO_2_-15 anodes consisted of splitting peaks of Pb 4f_7/2_ and Pb 4f_5/2_, which were fitted into four peaks at about 137.0, 137.9, 141.9 and 142.8 eV, indicating presence of Pb^4+^ and Pb^2+^ ions in PbO_2_ films [53, 54]. The peaks at 137.1 eV and 141.9 eV are attributed to Pb (IV), in agreement with the spectral values for PbO_2_ [55, 56], while the peaks at 137.9 eV and 142.8 eV are assigned to the binding energy of Pb (II). This demonstrates the presence of PbO or Pb_3_O_4_ (2PbO·PbO_2_) compounds in the 3DG-PbO_2_ and Y/3DG-PbO_2_-15 films [57]. Y 3d spectrum of the Y/3DG-PbO_2_-15 anode is displayed in Fig. 2(c). Two main components attributed to Y 3d_5/2_ and Y 3d_3/2_ can be observed at the binding energies of 156.9 and 159.2 eV, respectively, meaning that Y was doped into the β-PbO_2_ film in the state of Y_2_O_3_ [53, 58]. As shown in Fig. 2(d and e), the high-resolution O 1s XPS spectra of 3DG-PbO_2_ and Y/3DG-PbO_2_-15 anodes were fitted into three characteristic peaks, in which the peaks at 529.0 eV (Fig. 2(d)) and 529.1 eV (Fig. 2(e)) are ascribed to the lattice oxygen (O_L_) [59], the peaks of 530.9 eV (Fig. 2(d)) and 530.6 eV (Fig. 2(e)) are indexed to the adsorbed hydroxyl oxygen (O_OH_) species [42, 60], and the peaks of 532.6 eV (Fig. 2(d)) and 531.8 eV (Fig. 2(e)) are attributed to the adsorbed H_2_O (O_H2O_) [61]. In the electrocatalytic oxidation process, the hydroxyl groups can be transformed to •OH radicals. In general, a high proportion of O_OH_ species favors the production of more •OH radicals. Thus, the proportions of different oxygen species were calculated according to their peak areas. As a result, the proportions of O_L_, O_OH_, and O_H2O_ in the Y/3DG-PbO_2_-15 film were 44.19%, 35.86%, and 28.85%, while those in the 3DG-PbO_2_ film were 31.86%, 36.39%, and 31.75%, respectively. This result indicates that doping Y cannot improve the content of O_OH_ in the 3DG-PbO_2_ film.

### Electrochemical characterization

**Fig. 3:**
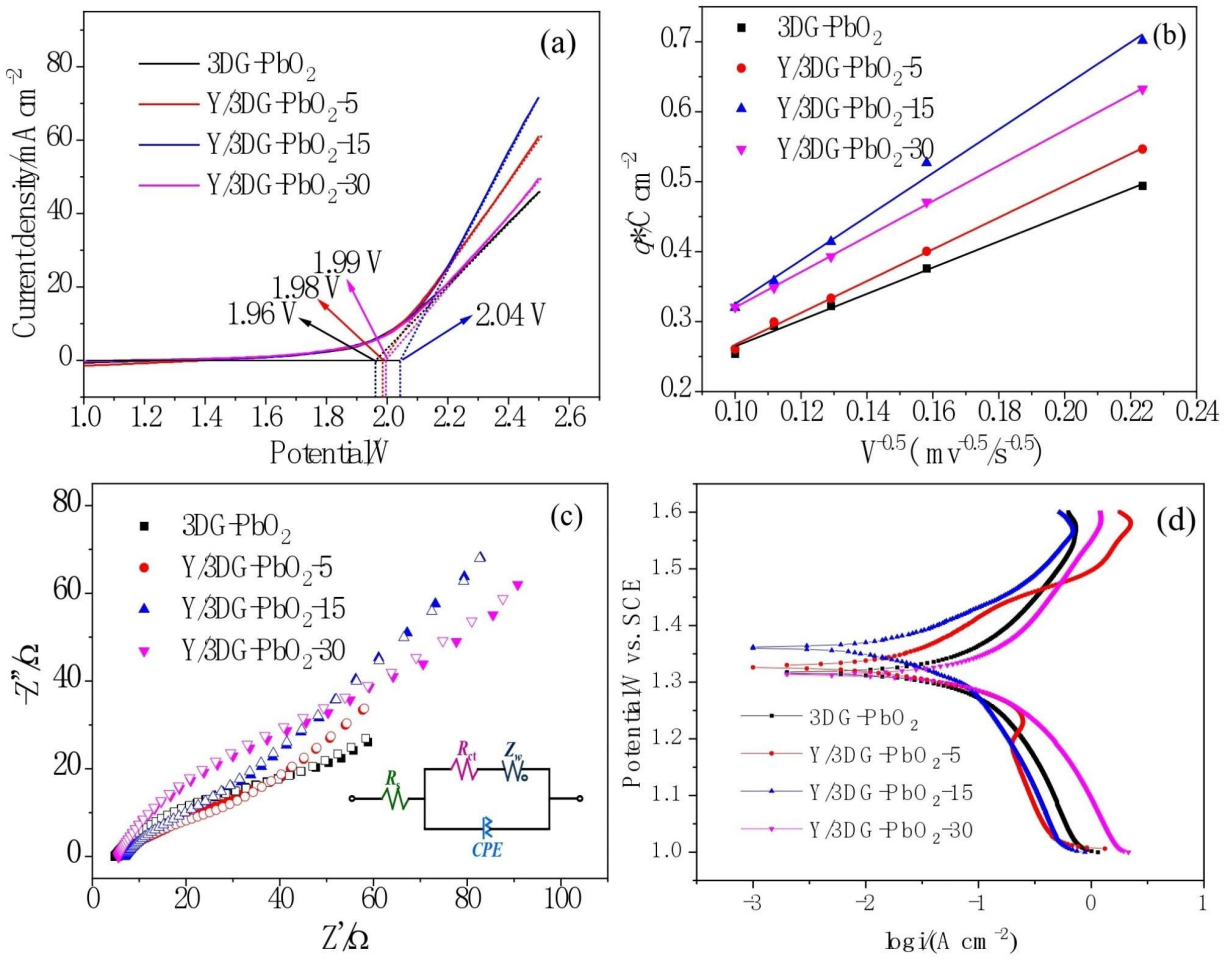
LSV curves **(a)**, q* against for v^− 1/2^**(b)**, Nyquist diagrams **(c)**, and Tafel curves **(d)** of 3DG-PbO_2_ and Y/3DG-PbO_2_ electrodes measured in 0.5 mol/L H_2_SO_4_ solution

In the process of electrochemical oxidation, hydroxyl (·OH) radicals are continuously generated on the surface of the anode, which plays the most key role in the degradation of pollutants. The oxygen evolution potential (OEP) is one of the important electrochemical indicators to evaluate the ability of hydroxyl radical generation. In general, high oxygen evolution potential (OEP) can inhibit the occurrence of oxygen evolution reaction, being beneficial to the accumulation of more ·OH radicals [54, 62, 63]. Thus, the OEP values of the as-prepared anodes were measured by LSV. As shown in Fig. 3(a), the OEP of the Y/3DG-PbO_2_-15 anode is 2.04 V, higher than those of 3DG-PbO_2_ (1.96 V), Y/3DG-PbO_2_-5 (1.98 V) and Y/3DG-PbO_2_-30 (1.99 V) anodes. Therefore, the OEP of the 3DG-PbO_2_ anode was increased by doping Y^3+^. The electrochemical activity of the anode is associated with the surface active sites of an electrode, which can be assessed by the voltammetric charge (q*) [37]. In general, anodes with higher q* have higher electrocatalytic activity [64]. Thus, CV tests were conducted in 0.5 M H_2_SO_4_ solution at scan rates of 20, 40, 60, 80, and 100 mV/s to assess the electrochemical activity of the prepared anodes. The obtained CV voltammograms are shown in Fig. S1 of supporting materials (SM). It can be observed that all the CV curves yielded a couple of redox peaks, which are ascribed to the reversible Pb^2+^/Pb^4+^ reaction [56, 65]. The q* values of all the CV curves were calculated using Eq. (4).

4 $$q*=\frac{\int idV}{v}$$

where $\int IDV$ is the integrated area of the CV curve and *v* is the scan rate. As displayed in Fig. 3(b), the q* values of four anodes followed the order of Y/3DG-PbO_2_-15 > Y/3DG-PbO_2_-30 > Y/3DG-PbO_2_-5 > 3DG-PbO_2_, suggesting that doping Y promoted the number of active sites of the 3DG-PbO_2_ anode and the Y/3DG-PbO_2_-15 electrode possessed more active sites than other three anodes. The influence of Y on the charge transfer resistance of the 3DG-PbO_2_ electrode was analyzed by EIS in 0.5 M H_2_SO_4_ at open circuit potential. Figure 3(c) shows the Nyquist diagrams of 3DG-PbO_2_, Y/3DG-PbO_2_-5, Y/3DG-PbO_2_-15, and Y/3DG-PbO_2_-30 electrodes. To obtain the charge transfer resistance of four electrodes, the Nyquist diagrams were fitted by the equivalent circuit model (the inset of Fig. 3(c)), where *R*_S_, *R*_CT_, *Z*_W_, and *CPE* represent the solution resistance, charge transfer resistance at the solution/electrode surface, Warburg impedance, and the constant phase element, respectively. The *R*_ct_ values of Y/3DG-PbO_2_-5 and Y/3DG-PbO_2_-15 electrodes were 33.54 and 27.22 Ω/cm^2^, respectively, lower than that of the 3DG-PbO_2_ electrode (45.31 Ω/cm^2^), indicating that doping Y reduced the charge transfer resistance during the electrochemical reaction process. However, the *R*_ct_ value of the Y/3DG-PbO_2_-30 electrode was 61.13 Ω/cm^2^, higher than that of the 3DG-PbO_2_ electrode. This may be related to the uneven surface of the Y/3DG-PbO_2_-30 electrode.

To investigate the influence of doping Y on the stability of the 3DG-PbO_2_ anode, a potentiodynamic polarization test was carried out in 0.5 M H_2_SO_4_ solution with the scan potential range from 1.0 to 1.6 V vs. SCE at a scan rate of 0.166 mV/s. The obtained Tafel curves are shown in Fig. 3d. The self-corrosion potential and self-corrosion current density were obtained by fitting the curves in the strong polarization region, which were (1.317 V, 68.76 µA/cm^2^), (1.326 V, 22.70 µA/cm^2^), (1.360 V, 47.15 µA/cm^2^), and (1.315 V, 11.78 µA/cm^2^) for 3DG-PbO_2_, Y/3DG-PbO_2_-5, Y/3DG-PbO_2_-15, and Y/3DG-PbO_2_-30, respectively. Compared to the 3DG-PbO_2_ electrode, the Y/3DG-PbO_2_-5 and Y/3DG-PbO_2_-15 electrodes possessed higher self-corrosion potential and lower self-corrosion current density, indicating that doping Y promoted the stability of the 3DG-PbO_2_ electrode [60, 66]. The higher stability of Y/3DG-PbO_2_-5 and Y/3DG-PbO_2_-15 was attributed to their more compact surface, which effectively prevented the electrolyte from entering the inside of the PbO_2_ film [67]. However, it also can be found that the Y/3DG-PbO_2_-30 electrode had lower self-corrosion potential and higher self-corrosion current density than the 3DG-PbO_2_ electrode, meaning that doping excessive Y would cause the decrease in compactness of the PbO_2_ film and the reduction the stability of 3DG-PbO_2_ electrode.

### *Generation of reactive oxidants*

**Fig. 4:**
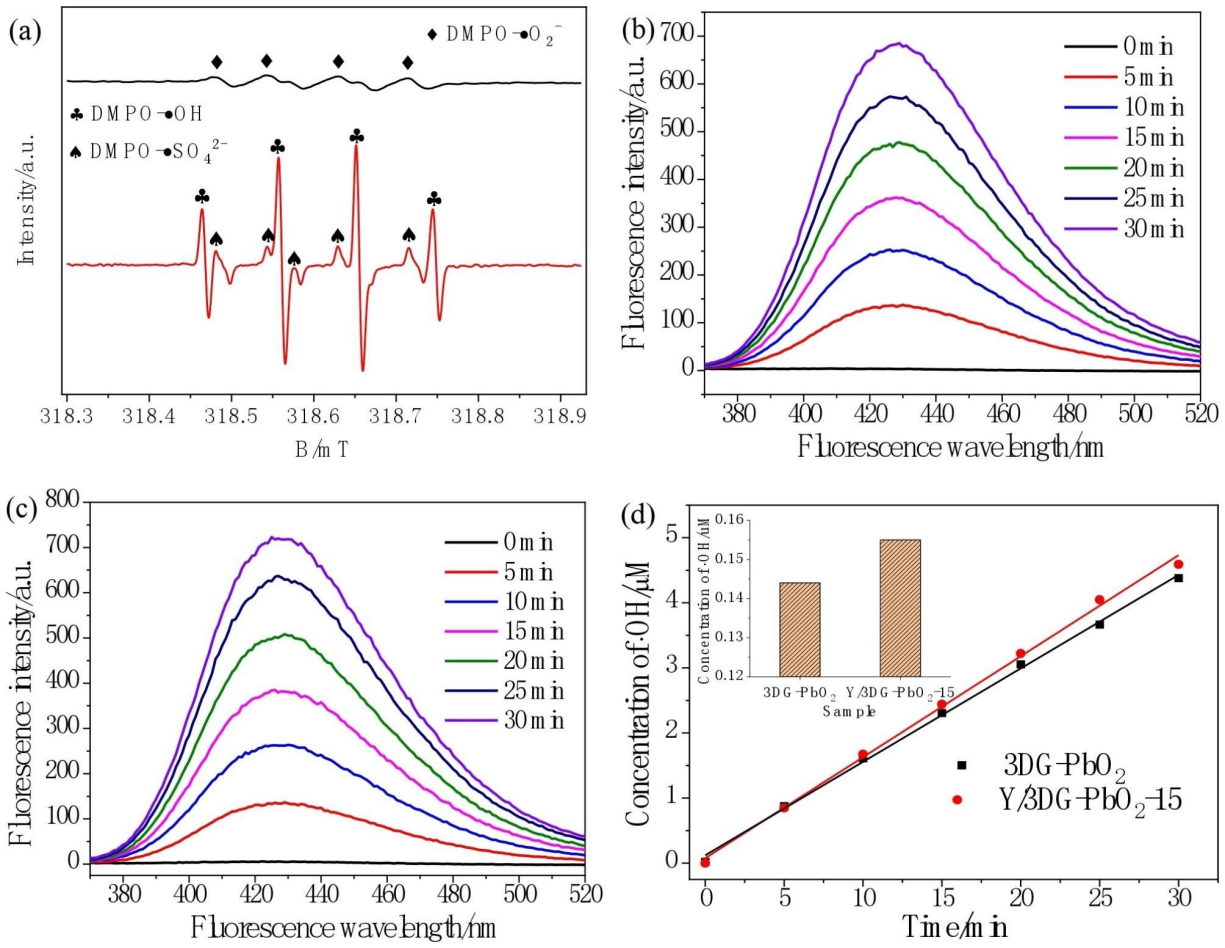
ESR spectra of the free radicals trapped by DMPO in electrochemical oxidation process with Y/3DG-PbO_2_-15 anode **(a)**, fluorescent spectra of electrolysis solution of 3DG-PbO_2_**(b)** and Y/3DG-PbO_2_-15 **(c)** anodes, and evolution of •OH radical concentration with electrolysis time for 3DG-PbO_2_ and Y/3DG-PbO_2_-15 anodes **(e)**

The reactive oxidants are responsible for the degradation of organic pollutants in the electrochemical oxidation process [68, 69]. The ESR technique was applied to reveal the possible reactive oxidant species formed in the electrochemical system with Y/3DG-PbO_2_-15 anode, in which the DMPO was used as spin-trapping reagent of •OH, •SO_4_^2–^ and •O_2_^–^ radicals. As shown in Fig. 4(a), no distinctive DMPO-•O_2_^–^ signal was observed in this electrochemical system. Instead, the strong characteristic patterns of DMPO-•OH with quartet signals of an intensity ratio of 1:2:2:1 were observed, confirming the generation of •OH radicals [70]. The DMPO-•SO_4_^2–^ signals were also observed in Fig. 4(a), but their intensity was lower than those of DMPO-•OH, meaning production of less •SO_4_^2–^ radicals. Therefore, the •OH radical is the predominant reactive oxidizing species in this electrochemical oxidation process. To analyze the influence of doping Y on the •OH radical generation performance of the 3DG-PbO_2_ anode, the TA was used as a quenching agent to measure the •OH radical generation amount in the 3DG-PbO_2_ and Y/3DG-PbO_2_-15 systems, in which the TA can quickly react with •OH radicals to form a fluorescent product of 2-HA [71]. As shown in Fig. 4(b and c), the fluorescent peak appeared at around 425 nm and its intensity gradually increased with extending electrolysis time for both 3DG-PbO_2_ and Y/3DG-PbO_2_-15 anodes, demonstrating that the •OH radicals generated continuously on the surface of these two anodes. Figure 4(d) provides the linear relations between the concentration of •OH radicals and electrolysis time. The higher rate constant of •OH radical generation (*k*_OH_) of 0.155 µM/min was achieved by the Y/3DG-PbO_2_-15 anode than that of the 3DG-PbO_2_ anode (0.144 µM/min), demonstrating that doping Y promoted the •OH radical generation ability of 3DG-PbO_2_ anode, and the Y/3DG-PbO_2_-15 should have higher electrocatalytic activity for degrading PFOS. Combined with the above XPS result that the Y didn’t improve the content of O_OH_ in the 3DG-PbO_2_ film, it can be concluded that the improvement of •OH radical generation ability of 3DG-PbO_2_ anode by doping Y was mainly due to the improvement of the OEP from 1.96 to 2.04 V.

### *Electrochemical oxidation of PFOS*

**Fig. 5:**
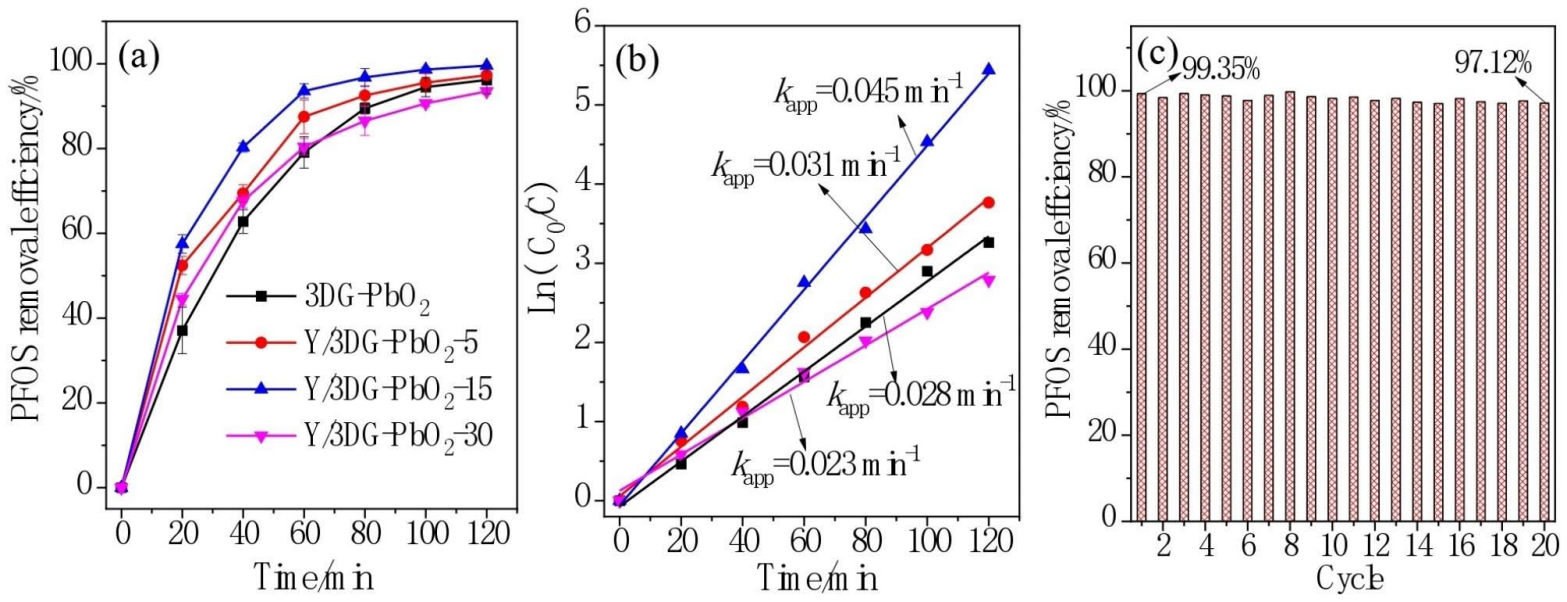
Variation curves of PFOS removal efficiency at different anodes **(a)**, kinetic analysis curves **(b)**, and electrochemical degradation of PFOS for twenty successive cycles using Y/3DG-PbO_2_-15 anode **(c)**

The electrocatalytic performance of 3DG-PbO_2_, Y/3DG-PbO_2_-5, Y/3DG-PbO_2_-15, and Y/3DG-PbO_2_-30 anodes for degrading PFOS is presented in Fig. 5. Figure 5(a) shows the removal efficiency of PFOS on four anodes with the extension of electrolysis time. It can be observed that the Y/3DG-PbO_2_-15 anode exhibited excellent catalytic performance and achieved 99.57% of PFOS removal efficiency, followed by Y/3DG-PbO_2_-5 (97.32%), 3DG-PbO_2_ (96.17%), and Y/3DG-PbO_2_-30 (93.51%) anodes. Figure 5(b) provides the results obtained from the kinetic analysis. The electrocatalytic oxidation process of PFOS can be well described by the pseudo-first-order kinetic equation, and the pseudo first order rate constants (*k*_app_) of 3DG-PbO_2_, Y/3DG-PbO_2_-5, Y/3DG-PbO_2_-15, and Y/3DG-PbO_2_-30 anodes were 0.028. 0.031, 0.045, and 0.023 min^–1^, respectively. The electrochemical oxidation of PFOS has also been reported in some literature. Zhuo et al. prepared a Ti/TiO_2_-NTs/Ag_2_O/PbO_2_ anode for electrochemical degradation of PFOS, and 74.87% of PFOS (90 mL of 0.0929 mM) degradation ratio was obtained after 180 min of electrolysis [72]. Zhuo and coworkers also reported an 89% PFOS removal ratio on a Ti/SnO_2−_Sb_2_O_3_/PbO_2_-PTFE anode after 3 h of electrochemical treatment [73]. 30% and 99% PFOS (20µM) were removed after 4 and 14 h electrolysis in an electrochemical oxidation process on a Ti_4_O_7_ anode, respectively [48]. The high removal efficiency of 99.57% after 120 min of electrolysis obtained by Y/3DG-PbO_2_-15 anode was significantly higher than those reported in the above literature. To compare the energy efficiency of PFOS degradation at the Y/3DG-PbO_2_ anodes with that reported in previous study [48], the EC values were calculated by Eq. (3) to be 16.6, 13.9, 10.5, and 19.1 kWh/m^3^ for 3DG-PbO_2_, Y/3DG-PbO_2_-5, Y/3DG-PbO_2_-15, and Y/3DG-PbO_2_-30 anodes, respectively with corresponding average cell potential of 5.4, 5.3, 5.1, and 5.2 V. It can be found that the EC (10.5 kWh/m^3^) of Y/3DG-PbO_2_-15 anode was significantly lower than ~ 20 kWh/m^3^ of Micro-Ti_4_O_7_ anode at the cell potential of ~ 5 V [48].

To evaluate the mineralization degree of PFOS with Y/3DG-PbO_2_ anodes, the TOC values of PFOS degradation solution were also measured and TOC removal efficiencies were calculated. After 120 min of electrolysis, the TOC removal efficiencies were 69.13%, 73.62%, 80.89%, and 65.41% with 3DG-PbO_2_, Y/3DG-PbO_2_-5, Y/3DG-PbO_2_-15, and Y/3DG-PbO_2_-30 anodes, respectively. Higher TOC removal efficiencies of Y/3DG-PbO_2_-5 and Y/3DG-PbO_2_-15 anodes than those of 3DG-PbO_2_ anode demonstrated that doping Y greatly improved the mineralization ability of 3DG-PbO_2_ anode.

Above results confirmed that the Y/3DG-PbO_2_-15 anode had more outstanding electrocatalytic activity for degrading PFOS than the other three anodes. This could be explained by its higher OEP, more active sites, smaller charger transfer resistance, and stronger •OH radicals generation ability.

In addition to high catalytic activity, good reusability is one of the important requirements for the practical application of the PbO_2_ anode [74]. The reusability of the Y/3DG-PbO_2_-15 anode was evaluated using consecutive electrolysis of PFOS. Figure 5(c) presents the removal percentages of PFOS over the Y/3DG-PbO_2_-15 anode after 120 min of electrolysis in twenty cycles. It can be observed that the change in PFOS removal efficiency within 20 consecutive cycles of electrolysis was slight. The removal efficiency of PFOS of the 20th cycle was 97.12%, slightly lower than 99.35% of the first cycle, demonstrating excellent reusability of the Y/3DG -PbO_2_-15 anode. To assess the safety of electrochemical degradation of PFOS with Y/3DG -PbO_2_-15 anode, the concentration of Pb^2+^ ions in the electrolyte was measured by ICP. After 120 min of electrolysis, the concentration of Pb^2+^ ions was 0.004 mg/L, far below the 0.01 mg/L of the Drinking-water Quality of WHO [71], indicating the safety of this process.

The response surface methodology (RSM) was used to describe the interaction between the response value (PFOS removal efficiency) and operating parameters (current density, initial PFOS concentration, pH, and Na_2_SO_4_ concentration) assisted with the BOX-Behnken method. The independent variables and designed 29 experimental sets are listed in Table S1 and Table S2 of SM, respectively. To obtain a precise model, parameters with a p-value > 0.05 were excluded from the model [75]. Thus, the quadratic polynomial model for PFOS removal efficiency (%) was presented as *Eq. (5*).

PFOS removal efficiency (%) = 86.98 + 10.56* A* – 15.48*B* – 8.16* C* + 2.03*D* – 4.61*AD* – 7.63*A*^2^ – 5.71*B*^2^ – 3.85*C*^2^ (5).

**Fig. 6:**
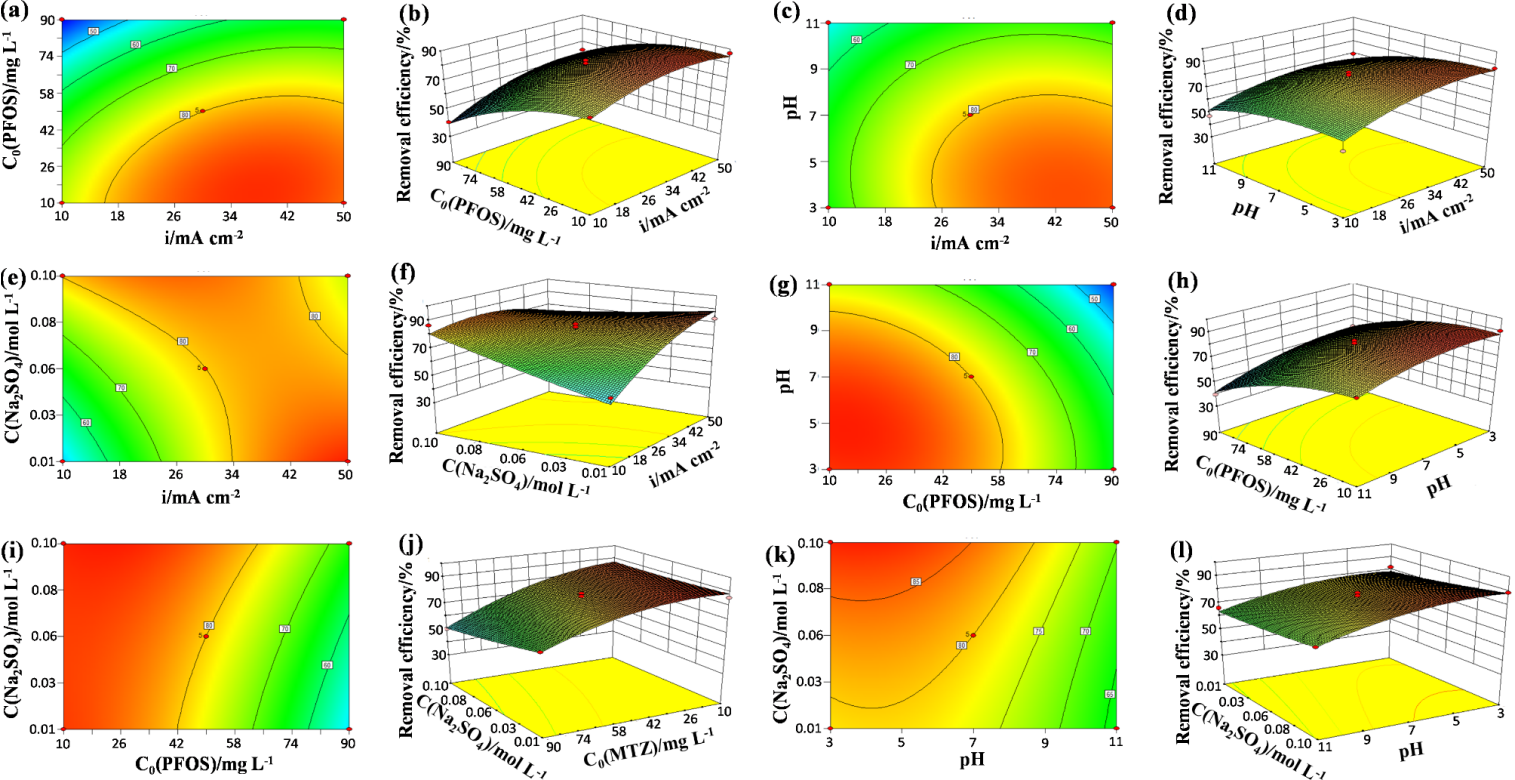
Contour and 3D response surface plots to show the interaction of different operation conditions for the PFOS degradation at Y/3DG-PbO_2_-15 anode

The ANOVA results are listed in Table S3. A large F-value of 19.63 and p-value < 0.0001 indicated the model was highly meaningful for fitting the actual data [76]. The values of *R*^2^ (0.9515) and adj-*R*^2^ (0.9030) were close to unity and the F-value and p-value for Lack of Fit are 4.00 and 0.0970, respectively, meaning the high correlation between the experimental and the predicted removal efficiency of PFOS [77]. The value of adequate precision (14.999) was larger than 4, indicating the signal-to-noise of the model was adequate. The coefficient of variation (C.V.) was 6.35, less than the critical value of 10%, implying the high reproducibility of the model. The F-values of current density, initial PFOS concentration, pH, and Na_2_SO_4_ concentration were 39.52, 120.67, 40.25, and 9.77, respectively, suggesting the effect of these variables on PFOS degradation efficiency followed the sequence of Initial PFOS concentration > Current density > pH > Na_2_SO_4_ concentration. The graphical 3D and 2D contour plots in Fig. 6 described the interaction of independent variables. PFOS degradation efficiency increased with the current density increasing from 10 to 50 mA/cm^2^, decreased from the initial PFOS concentration increasing from 10 to 90 mg/L. Higher Na_2_SO_4_ concentration in the range of 0.01–0.1 M and lower pH in the range of 3–11 were more conducive to the degradation of PFOS. Fig. S2 (a and b) of SM shows the plots of internally studentized residuals versus predicted response, internally studentized residuals versus experimental runs, and predicted response versus experimental value. It can be observed from Fig. S2(a and b) that all the residuals are scattered in the standard deviation range, representing a good accordance between the experimental value and predicted response [78]. The good linear relationship between the predicted and actual values (Fig. S2(c) of SM) also confirmed the adequation of the proposed model [79]. The optimization of electrochemical oxidation operation parameters was performed by setting the response (PFOS removal efficiency after 40 min of electrolysis, %) to maximize and setting the variables to within the working range. Table S4 of SM lists the provided solutions by the Box-Behnken methodology, the maximum PFOS degradation of 97.84% was acquired by the 42nd solution (current density = 50 mA/cm^2^, initial PFOS concentration = 12.21 mg/L, pH value = 5.39, Na_2_SO_4_ concentration = 0.01 M). The experiment was carried out under the optimized experimental conditions to evaluate the validity of the predicted model. The obtained experimental value of 97.16% was very close to the predicted value of 97.84%.

## Conclusion

In this work, a novel Y/3DG-PbO_2_ anode was successfully fabricated by introducing Y into the 3DG-PbO_2_ anode. Compared with the 3DG-PbO_2_ anode, the Y/3DG-PbO_2_-15 anode exhibited a denser film and smaller crystal size. At the same time, the Y/3DG-PbO_2_-15 anode also possessed higher oxygen evolution potential (2.04 V vs. SCE), more voltammetric quantity (0.70 C/cm^2^ at a scan rate of 20 mV/s), smaller charge transfer resistance (27.22 Ω/cm^2^), and larger •OH radical generation rate (0.155 µM/min) than those of 3DG-PbO_2_ anode (1.96 V vs. SCE, 0.49 C/cm^2^, 45.31 Ω/cm^2^ and 0.144 µM/min). As a result, the Y/3DG-PbO_2_-15 anode exhibited excellent electrocatalytic activity for the electrochemical degradation of PFOS. The rate constant of PFOS degradation over the Y/3DG-PbO_2_-15 anode was 0.045 min^–1^, significantly higher than 0.028 min^–1^ over the 3DG-PbO_2_ anode. According to the RSM, the optimum electrochemical oxidation conditions for PFOS degradation were current density = 50 mA/cm^2^, initial PFOS concentration = 12.21 mg/L, pH value = 5.39, and Na_2_SO_4_ concentration = 0.01 M. In this case, 97.16% of PFOS removal was acquired after 40 min of electrolysis, which was very close to the predicted value of 97.84%. Both of Tafel test and consecutive electrolysis experiments confirmed the excellent stability of the Y/3DG-PbO_2_-15 anode. In the Tafel test, the Y/3DG-PbO_2_-15 electrode obtained a higher self-corrosion potential (1.360 V) and lower self-corrosion current density (0.047 A) than the 3DG-PbO_2_ electrode (1.317 V, 0.069 A). In consecutive cycles of electrolysis, the removal percentages of PFOS over the Y/3DG-PbO_2_-15 anode after 20 cycles was still 97.12%, slightly lower than 99.35% of the first cycle.

### Electronic supplementary material

Below is the link to the electronic supplementary material.

Supplementary Material 1

## Data Availability

The datasets used and/or analyzed during the current study are available from the corresponding author on reasonable request.
