# Supplementary material for: Y-mediated optimization of 3DG-PbO2 anode for electrochemical degradation of PFOS
Source: BMC Chem. 2023 Oct 27;17(1):146. doi: 10.1186/s13065-023-01057-3 (PMC10612263; doi:10.1186/s13065-023-01057-3)
Supplement: Supplementary file 1 — Supplementary Material 1 [file 13065_2023_1057_MOESM1_ESM.docx]

**Supporting Materials**

**Y-mediated Optimization of 3DG-PbO_2_ Anode for Electrochemical Degradation of PFOS**

Xiaoyue Duan^a,b,c^, Ziqi Ning^b,c^, Weiyi Wang^a^, Yitong Li^b,c^, Xuesong Zhao^b,c^, Liyue Liu^c^, Wenqian Li^c^, Limin Chang^a,*^

*a Key Laboratory of Preparation and Applications of Environmental Friendly Materials (Jilin Normal University), Ministry of Education, Changchun 130103, China*

*b Key Laboratory of Environmental Materials and Pollution Control (Jilin Normal University), Education Department of Jilin Province, Siping 136000, China*

*c College of Engineering, Jilin Normal University, Siping, 136000, China*

Corresponding authors.

* E-mail address: [changlimin2139@163.com](mailto:changlimin2139@163.com) (L. Chang).

**Fig. S1. CV curves of 3DG-PbO_2_ (a),** **Y/3DG-PbO_2_-5 (b), Y/3DG-PbO_2_-15 (c) and Y/3DG-PbO_2_-30 (d).**

**Table S1.** Variables and levels of Box-Behnken experimental design.

| Variables | Code | Unit | Range and level | | |
| --- | --- | --- | --- | --- | --- |
|  |  |  | Low level (–1) | Central point (0) | High level (+1) |
| Current density | *A* | mA/cm^2^ | 10 | 30 | 50 |
| Initial PFOS concentration | *B* | mg/L | 10 | 50 | 90 |
| pH | *C* | - | 3 | 7 | 11 |
| Na_2_SO_4_ concentration | *D* | mol/L | 0.01 | 0.06 | 0.1 |

**Table S2.** The experimental design and results of response surface methodology.

| Run | A  (Current density, mA/cm^2^) | B  (Initial PFOS concentration, mg/L) | C  (pH) | D  (Concentration of Na_2_SO_4_, mol/L) | PFOS removal efficiency after 40 min of electrolysis, % |
| --- | --- | --- | --- | --- | --- |
| 1 | 50.00 | 50.00 | 3.00 | 0.06 | 87.21 |
| 2 | 30.00 | 90.00 | 7.00 | 0.10 | 66.85 |
| 3 | 10.00 | 10.00 | 7.00 | 0.06 | 76.06 |
| 4 | 30.00 | 50.00 | 11.00 | 0.01 | 66.32 |
| 5 | 10.00 | 50.00 | 7.00 | 0.01 | 54.38 |
| 6 | 30.00 | 50.00 | 11.00 | 0.10 | 70.35 |
| 7 | 10.00 | 50.00 | 7.00 | 0.10 | 86.27 |
| 8 | 30.00 | 90.00 | 11.00 | 0.06 | 39.65 |
| 9 | 30.00 | 90.00 | 3.00 | 0.06 | 63.19 |
| 10 | 10.00 | 50.00 | 11.00 | 0.06 | 47.22 |
| 11 | 30.00 | 10.00 | 7.00 | 0.10 | 85.77 |
| 12 | 30.00 | 10.00 | 7.00 | 0.01 | 83.54 |
| 13 | 50.00 | 50.00 | 7.00 | 0.10 | 70.94 |
| 14 | 50.00 | 10.00 | 7.00 | 0.06 | 87.08 |
| 15 | 50.00 | 90.00 | 7.00 | 0.06 | 64.27 |
| 16 | 30.00 | 10.00 | 11.00 | 0.06 | 75.97 |
| 17 | 30.00 | 50.00 | 7.00 | 0.06 | 80.86 |
| 18 | 30.00 | 50.00 | 7.00 | 0.06 | 76.67 |
| 19 | 30.00 | 50.00 | 7.00 | 0.06 | 81.12 |
| 20 | 30.00 | 50.00 | 7.00 | 0.06 | 83.11 |
| 21 | 10.00 | 90.00 | 7.00 | 0.06 | 40.35 |
| 22 | 30.00 | 90.00 | 7.00 | 0.01 | 50.41 |
| 23 | 30.00 | 50.00 | 3.00 | 0.01 | 81.93 |
| 24 | 30.00 | 10.00 | 3.00 | 0.06 | 90.15 |
| 25 | 50.00 | 50.00 | 11.00 | 0.06 | 69.49 |
| 26 | 30.00 | 50.00 | 7.00 | 0.06 | 78.04 |
| 27 | 30.00 | 50.00 | 3.00 | 0.10 | 88.79 |
| 28 | 10.00 | 50.00 | 3.00 | 0.06 | 58.13 |
| 29 | 50.00 | 50.00 | 7.00 | 0.01 | 82.91 |

**Table S3.** ANOVA analysis of the quadratic polynomial model.

| Number | Sum of squares | Mean square | *F*-value | *p*-value |  |
| --- | --- | --- | --- | --- | --- |
| Model | 5735.9 | 409.65 | 19.63 | < 0.0001 | significant |
| *A* | 824.86 | 824.86 | 39.52 | < 0.0001 |  |
| *B* | 2518.65 | 2518.65 | 120.67 | < 0.0001 |  |
| *C* | 840.01 | 840.01 | 40.25 | < 0.0001 |  |
| *D* | 204.02 | 204.02 | 9.77 | 0.0074 |  |
| *AB* | 41.60 | 41.60 | 1.99 | 0.1798 |  |
| *AC* | 11.59 | 11.59 | 0.56 | 0.4684 |  |
| *AD* | 480.92 | 480.92 | 23.04 | 0.0003 |  |
| *BC* | 21.90 | 21.90 | 1.05 | 0.3230 |  |
| *BD* | 50.48 | 50.48 | 2.42 | 0.1422 |  |
| *CD* | 2.00 | 2.00 | 0.096 | 0.7613 |  |
| *A*^2^ | 340.24 | 340.24 | 16.30 | 0.0012 |  |
| *B*^2^ | 352.33 | 352.33 | 16.88 | 0.0011 |  |
| *C*^2^ | 194.88 | 194.88 | 9.34 | 0.0086 |  |
| *D*^2^ | 3.91 | 3.91 | 0.19 | 0.6718 |  |
| Residual | 292.21 | 20.87 | - | - |  |
| Lack of Fit | 265.62 | 26.56 | 4.00 | 0.0970 | Not significant |
| Pure Error | 26.59 | 6.65 | - | - |  |
| Cor Total | 6027.30 | - | - | - |  |
| R^2^ = 0.9515 Adj R^2^ = 0.9030 C.V.% = 6.35 Adeq Precisior = 14.999 | | | | | |

**Table S4.** Proposed solutions.

| Number | A  (Current density, mA/cm^2^) | B  (Initial PFOS concentration, mg/L) | C  (pH) | D  (Concentration of Na_2_SO_4_, mol/L) | MTZ removal efficiency after 40 min of electrolysis, % | Desirability |
| --- | --- | --- | --- | --- | --- | --- |
| 1 | 50.00 | 26.01 | 7.65 | 0.01 | 92.6811 | 1.000 |
| 2 | 50.00 | 40.04 | 3.96 | 0.03 | 91.5041 | 1.000 |
| 3 | 50.00 | 21.11 | 7.21 | 0.02 | 91.5254 | 1.000 |
| 4 | 50.00 | 25.14 | 3.98 | 0.05 | 90.3511 | 1.000 |
| 5 | 50.00 | 17.69 | 3.89 | 0.02 | 94.8437 | 1.000 |
| 6 | 50.00 | 19.39 | 5.39 | 0.02 | 95.2494 | 1.000 |
| 7 | 50.00 | 14.99 | 4.40 | 0.02 | 95.0427 | 1.000 |
| 8 | 50.00 | 27.89 | 3.60 | 0.04 | 90.6595 | 1.000 |
| 9 | 50.00 | 21.02 | 4.20 | 0.05 | 90.4549 | 1.000 |
| 10 | 50.00 | 13.77 | 7.65 | 0.01 | 94.2777 | 1.000 |
| 11 | 50.00 | 12.61 | 6.44 | 0.04 | 90.4917 | 1.000 |
| 12 | 50.00 | 15.92 | 4.26 | 0.02 | 97.0271 | 1.000 |
| 13 | 50.00 | 30.33 | 4.44 | 0.03 | 92.8338 | 1.000 |
| 14 | 50.00 | 40.20 | 5.87 | 0.02 | 91.5707 | 1.000 |
| 15 | 50.00 | 30.40 | 7.56 | 0.01 | 91.9705 | 1.000 |
| 16 | 50.00 | 21.82 | 5.19 | 0.04 | 91.9836 | 1.000 |
| 17 | 50.00 | 24.69 | 6.95 | 0.03 | 90.2992 | 1.000 |
| 18 | 50.00 | 22.38 | 8.20 | 0.01 | 92.4836 | 1.000 |
| 19 | 50.00 | 42.94 | 3.08 | 0.03 | 90.8729 | 1.000 |
| 20 | 50.00 | 21.71 | 5.11 | 0.04 | 90.6764 | 1.000 |
| 21 | 50.00 | 31.36 | 8.25 | 0.01 | 90.2962 | 1.000 |
| 22 | 50.00 | 19.86 | 4.68 | 0.04 | 90.458 | 1.000 |
| 23 | 50.00 | 29.51 | 3.28 | 0.03 | 92.6914 | 1.000 |
| 24 | 50.00 | 50.65 | 4.42 | 0.02 | 90.5143 | 1.000 |
| 25 | 50.00 | 21.74 | 3.36 | 0.04 | 92.0512 | 1.000 |
| 26 | 50.00 | 54.05 | 3.02 | 0.02 | 90.245 | 1.000 |
| 27 | 50.00 | 14.30 | 6.43 | 0.03 | 91.3275 | 1.000 |
| 28 | 50.00 | 10.06 | 8.10 | 0.02 | 90.1826 | 1.000 |
| 29 | 50.00 | 21.61 | 4.48 | 0.01 | 97.1427 | 1.000 |
| 30 | 50.00 | 29.16 | 3.26 | 0.04 | 90.3879 | 1.000 |
| 31 | 50.00 | 30.55 | 4.71 | 0.04 | 90.8206 | 1.000 |
| 32 | 50.00 | 10.81 | 3.75 | 0.04 | 90.8938 | 1.000 |
| 33 | 50.00 | 53.67 | 4.09 | 0.01 | 91.0922 | 1.000 |
| 34 | 50.00 | 22.99 | 8.79 | 0.01 | 90.3011 | 1.000 |
| 35 | 50.00 | 30.94 | 6.67 | 0.03 | 90.5196 | 1.000 |
| 36 | 50.00 | 17.01 | 4.94 | 0.03 | 94.0723 | 1.000 |
| 37 | 50.00 | 24.60 | 4.63 | 0.04 | 90.6125 | 1.000 |
| 38 | 50.00 | 26.28 | 8.58 | 0.01 | 90.7952 | 1.000 |
| 39 | 50.00 | 12.17 | 6.17 | 0.03 | 93.0672 | 1.000 |
| 40 | 50.00 | 22.95 | 6.06 | 0.04 | 90.4325 | 1.000 |
| 41 | 50.00 | 17.98 | 3.88 | 0.04 | 91.3811 | 1.000 |
| 42 | 50.00 | 12.21 | 5.39 | 0.01 | 97.8423 | 1.000 |
| 43 | 50.00 | 20.27 | 4.21 | 0.04 | 92.1301 | 1.000 |
| 44 | 50.00 | 49.08 | 5.87 | 0.01 | 90.4638 | 1.000 |
| 45 | 50.00 | 44.41 | 5.77 | 0.02 | 91.6014 | 1.000 |
| 46 | 50.00 | 20.68 | 7.73 | 0.01 | 93.3986 | 1.000 |
| 47 | 50.00 | 23.80 | 4.29 | 0.02 | 96.6949 | 1.000 |
| 48 | 50.00 | 46.02 | 3.39 | 0.03 | 90.7597 | 1.000 |
| 49 | 50.00 | 34.32 | 3.58 | 0.04 | 90.9569 | 1.000 |
| 50 | 49.91 | 10.02 | 5.41 | 0.04 | 90.1531 | 0.999 |
| 51 | 50.00 | 10.00 | 3.31 | 0.05 | 89.3565 | 0.992 |
| 52 | 50.00 | 47.91 | 3.00 | 0.04 | 88.816 | 0.987 |
| 53 | 48.38 | 40.16 | 3.74 | 0.04 | 90.15 | 0.979 |
| 54 | 50.00 | 30.28 | 3.10 | 0.08 | 84.1865 | 0.939 |
| 55 | 50.00 | 39.34 | 10.79 | 0.01 | 78.8485 | 0.881 |

**Fig. S2**. Plots of residuals vs. predicted (a), residuals vs. run number (b) and predicted vs. actual (c).
